# TIGIT Blockade Exerts Synergistic Effects on Microwave Ablation Against Cancer

**DOI:** 10.3389/fimmu.2022.832230

**Published:** 2022-03-07

**Authors:** Yaping Chen, Hao Huang, Yuan Li, Wenlu Xiao, Yingting Liu, Rongzhang Chen, Yulan Zhu, Xiao Zheng, Changping Wu, Lujun Chen

**Affiliations:** ^1^ Department of Tumor Biological Treatment, The Third Affiliated Hospital of Soochow University, Changzhou, China; ^2^ Jiangsu Engineering Research Center for Tumor Immunotherapy, The Third Affiliated Hospital of Soochow University, Changzhou, China; ^3^ Institute of Cell Therapy, The Third Affiliated Hospital of Soochow University, Changzhou, China; ^4^ Department of Oncology, The Third Affiliated Hospital of Soochow University, Changzhou, China

**Keywords:** ablation, immune checkpoint inhibitors, TIGIT, immunotherapy, tumor microenvironment

## Abstract

**Background:**

Combination immunotherapy based on immune checkpoint inhibitors (ICIs) has shown great success in the treatment of many types of cancers and has become the mainstream in the comprehensive treatment of cancers. Ablation in combination with immunotherapy has achieved tremendous efficacy in some preclinical and clinical studies. To date, our team proved that ablation in combination with ICIs was a promising antitumor therapeutic strategy for the liver metastasis of colorectal cancer (CRC). Moreover, we found that the expression of T cell immunoglobulin and immunoreceptor tyrosine-based inhibitory motif domain (TIGIT) expression was up-regulated after microwave ablation (MWA), indicating that TIGIT was involved in immunosuppression, and the combination of MWA and TIGIT blockade represented a potential clinical treatment strategy.

**Methods:**

In the present study, we examined the expression of TIGIT using a preclinical mouse model treated with MWA. Moreover, we evaluated the antitumor functions of MWA alone or in combination with TIGIT blockade by monitoring tumor growth and survival of the mice. Besides, we also detected the numbers of tumor-infiltrating lymphocytes (TILs), and effector molecules of CD8^+^ T cells using flow cytometry. Finally, we analyzed the single-cell RNA sequencing (scRNA-seq) data from the MWA and MWA plus anti-TIGIT groups.

**Results:**

The expression of TIGIT in various immune cells was up-regulated after MWA, and the addition of TIGIT blockade to MWA prolonged survival and delayed tumor growth in the MC38 tumor model. Taken together, our findings showed that TIGIT blockade in combination with MWA significantly promoted the expansion and functions of CD8^+^ TILs and reshaped myeloid cells in the tumor microenvironment (TME) using flow cytometry and scRNA-seq analysis.

**Conclusions:**

TIGIT blockade in combination with MWA was a novel treatment strategy for the liver metastasis of CRC, and this combination therapy could reprogram the TME toward an antitumor environment.

## Introduction

As a local, minimally invasive treatment guided by ultrasound or computed tomography, tumor ablation, including radiofrequency ablation (RFA) and microwave ablation (MWA), is widely used in the treatments for many human solid tumors (1–3). Accumulating evidence has found that tumor ablation is safe and feasible for hepatocellular carcinoma (HCC), liver metastasis of colorectal cancer (CRC), renal cancer, prostate cancer, osteoid osteoma, thoracic cancer, and lung cancer, *etc* (4–8). Ablation not only induces necrosis to destroy cancer tissue but also triggers antigen release and even immune responses (2, 9). We have previously reported that ablation can induce immune responses against tumors, and the programmed death-ligand 1/programmed death 1 (PD-L1/PD-1) axis plays an important role in attenuating RFA-induced antitumor immunity (10). However, ablation does not always induce long-term anti-tumor immunity, and in some cases, it can also lead to immunosuppression in the late stage due to elevated expression of PD-1 on T cells, PD-L1 on antigen-presenting cells or tumor cells (10). Moreover, we have also proved the mechanism that the incomplete RFA is involved in the rapid tumor progression, thus hindering the PD-1 blockade immunotherapy due to the production of CCL2 by cancer cells (11).

In addition to surgery, chemotherapy, and radiotherapy, treatment with immune checkpoint inhibitors (ICIs) is another effective strategy for the treatment of cancers (12). Since the cytotoxic T-lymphocyte-associated antigen 4 (CTLA-4) antibody ipilimumab has become the first ICI used in melanoma therapy, anti-PD-L1/PD-1 agents have been approved for the treatment of melanoma, non-small-cell lung cancer, head and neck squamous cell carcinoma, MMS-H/dMMR solid tumors, Hodgkin’s lymphoma, and other cancers (13–17). However, only a small subset of patients achieve a good clinical response. These results indicate the presence of another immunosuppressive signal in the TME. T cell immunoglobulin and immunoreceptor tyrosine-based inhibitory motif domain (TIGIT), which is also known as Vsig9, Vstm3, or WUCAM, is a coinhibitory receptor similar to PD-1, lymphocyte activation gene-3 (LAG-3), and T cell immunoglobulin-3 (TIM-3) (18). TIGIT has three ligands, including CD155 [poliovirus receptor (PVR)], CD112 [nectin-2 or poliovirus receptor-related 2 (PVRL2)], and CD113 (nectin-3 or PVRL3), and it has a higher affinity for CD155 (19). TIGIT is expressed on T cells and natural killer (NK) cells, and it inhibits the activation of these cells (19–21). TIGIT inhibits T cells by competing with CD226 for binding to the same PVR ligand (22). Multiple studies have found that TIGIT may regulate the effector function of antitumor and antiviral CD8^+^ T cells effector function *via* participating in the TIGIT-CD96-CD112R-CD226 axis in the cancer immunotherapy (23). Furthermore, according to a recent study, TIGIT is expressed on tumor-infiltrating NK cells, and TIGIT blockade reverses the exhaustion of NK cells independently of the adaptive immune system, namely, CD8^+^ T cell-mediated tumor reactivity (24). Importantly, TIGIT blockade can reduce the suppressive function of Foxp3^+^ regulatory T cells (Tregs) (25). In addition, several ongoing clinical trials targeting TIGIT as the treatment of advanced solid cancers including non-small-cell lung cancer (such as NCT03119428, NCT02913313, NCT03563716, and NCT02794571), either as a single agent or in combination with other ICIs, are being performed (26). According to the phase I and II trials, the TIGIT inhibitor tiragolumab alone or in combination with the PD-L1 inhibitor atezolizumab, has achieved statistically significant results in the treatment of multiple solid malignancies, most notably non-small cell-lung cancer (27, 28). Therefore, TIGIT blockade has been a promising strategy for antitumor immunotherapy.

Studies have found that MWA provides several advantages over the other forms of thermal ablation. Specifically, MWA induces larger volumes of necrosis, achieves faster ablation rates, and results in greater sphericity of the necrotic area, which can ablate larger nodules (29, 30). Therefore, we used MWA to treat mice with MC38 colon cancer and assessed the immune response. In the present study, the expression of TIGIT was up-regulated after MWA. This result indicated that the expression of TIGIT, which was up-regulated as an immunosuppressive signal after MWA, had important implications in combination therapy. Meanwhile, the addition of TIGIT blockade to MWA prolonged survival and delayed tumor growth in the mouse MC38 tumor model. Overall, TIGIT blockade in combination with MWA promoted the expansion and functions of CD8^+^ T cells and reshaped myeloid cells by recruiting CD8^+^ TILs infiltrating into TME. These results supported the notion that TIGIT blockade in combination with MWA could serve as a novel therapeutic strategy and could synergistically improve the anti-tumor immunity.

## Results

### High Expression of TIGIT in TILs and Changes in Distant Tumors After MWA

**Figure 1:**
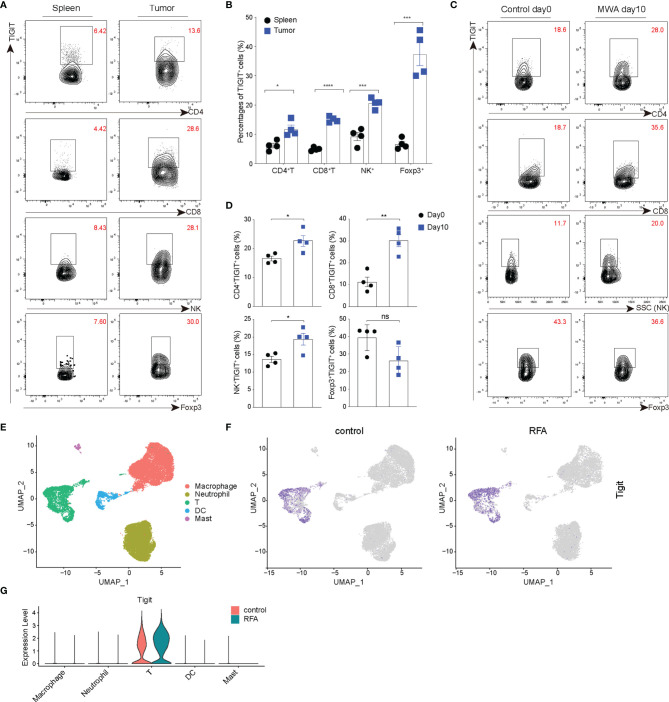
TIGIT is up-regulated in the distant tumors after MWA treatment and expressed at high levels in TILs. **(A, B)** A total of 1.5×10^6^ MC38 cells were inoculated subcutaneously into the left flank of C57BL/6 mice (n=4). **(A)** Representative flow cytometry plots of TIGIT expression in spleens and TILs of MC38 tumor-bearing mice (not subjected to MWA). **(B)** Quantitative percentage of TIGIT in TILs and spleens of MC38 tumor-bearing mice (not subjected to MWA). **(C, D)** A total of 3×10^6^ MC38 cells were subcutaneously inoculated into the bilateral flanks of C57BL/6 mice (n=4). MWA was performed on the tumor on the right flank when the tumor volume reached approximately 300 mm^3^. **(C)** Representative flow cytometry plots of TIGIT expression in TILs, including CD4^+^ T, CD8^+^ T, NK cells and CD4^+^ Foxp3^+^ Tregs on day 0 after MWA and day 10 after MWA. **(D)** Quantitation of the percentage of TIGIT in TILs on day 0 before MWA and day 10 of MWA. **(E–G)** Cluster analysis. Immune cells from vaccinated Panc02 tumor-bearing mice belonging to the control group (not subjected to RFA) and RFA group were determined by performing a UMAP dimensionality reduction analysis of scRNA-seq data and were colored according to the expression of TIGIT. Data were presented as the mean ± SEM, n=4, ns (not significant, *P*>0.05), * *P*<0.05, ** *P*<0.01, *** *P*<0.001, and **** *P*<0.0001 according to two-tailed unpaired Student’s *t*-test.

TIGIT is a coinhibitory receptor, and its high expression is consistent across multiple types of solid tumors (23). We assessed the expression of TIGIT in TILs using the MC38 tumor model, and the expression of TIGIT was significantly higher in TILs, including CD4^+^ TILs, CD8^+^ TILs, and NK cells, compared with the spleen (**Figures 1A, B**). Next, we examined the expression of TIGIT in the distant tumor environment following MWA treatment in the MC38 colon cancer model. The MWA treatment was performed as described as in the *Materials and Methods*. On day 10 after MWA, the expression of TIGIT was markedly increased in CD4^+^ TILs, CD8^+^ TILs, and NK cells, while it was not significantly changed in Tregs (**Figures 1C, D**). We also compared the expression of TIGIT on day 10 after MWA with untreated animals at day 10 (Data shown in **Supplementary Figure 1**). We found there were no significant difference in the expression of TIGIT in TILs of untreated animals on day 0 compared with day 10. And the expression of TIGIT in TILs, including CD4^+^ TILs, CD8^+^ TILs, and NK cells was upregulated on day 10 after MWA compared with the group of untreated animals on day 10, while it was not significantly changed in Tregs. Based on these results, local MWA enhanced anti-tumor immune responses in distant tumors. And the tumor progression does not trigger changes in the immune landscape related to TIGIT upregulation. Consistently, we found the expression of TIGIT was also upregulated in T cells after ablation by analyzing the published scRNA-seq data from the pancreatic ductal adenocarcinoma (PDAC) mouse model (**Figures 1E–G**) (31).

### The Combination of MWA and Anti-TIGIT Treatment Synergistically Inhibits the Growth of Distant Tumors

**Figure 2:**
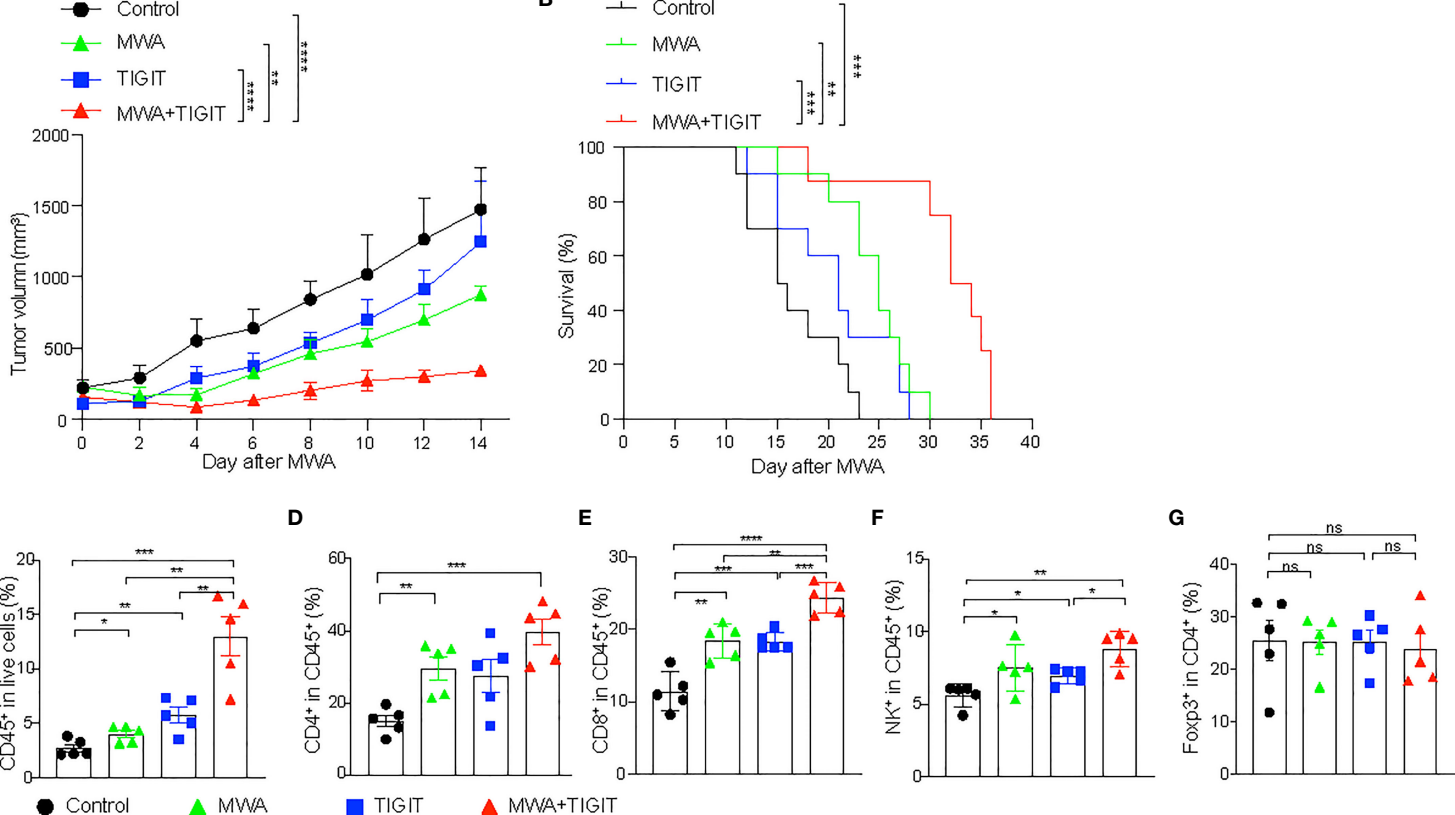
The combination of MWA and anti-TIGIT treatment synergistically inhibits the growth of distant tumors. **(A–G)** A total of 3×10^6^ MC38 cells were subcutaneously inoculated into the bilateral flanks of C57BL/6 mice. Mice with MC38 colon cancer mice were divided into four groups: the control group, which was treated with an isotype control antibody; the MWA group, which was treated with MWA and an isotype control antibody; the TIGIT group, which was treated with anti-TIGIT; and the MWA plus TIGIT group, which was treated with MWA and anti-TIGIT. MWA was conducted only for the tumor on the right flank when the tumor volume reached approximately 300 mm^3^ and the right flank tumor was cured after MWA. Anti-TIGIT mAbs (200 μg per mouse per injection) were intraperitoneally administered to the mice on day 1 after MWA, and subsequently once every 3 days for a total of four injections. The mice were considered dead when the tumor size reached 20mm in diameter or tumor ruptured. **(A)** The tumor size of on the left flank was measured every 2 days after MWA. **(B)** Kaplan-Meier survival curves were shown (n=10). **(C)** Frequencies of infiltrating CD45^+^ TILs (n=5). **(D)** Frequencies of infiltrating CD4^+^ TILs (n=5). **(E)** Frequencies of infiltrating CD8^+^ TILs (n=5). **(F)** Frequencies of infiltrating NK^+^ cells (n=5). **(G)** Frequencies of infiltrating Tregs (n=5). Data were presented as the mean ± SEM, ns (not significant, *P*>0.05), * *P*<0.05, ** *P*<0.01, *** *P*<0.001, and **** *P*<0.0001 according to the two-way ANOVA test and the log-rank test **(A, B)**, and the one-way ANOVA test **(C–G)**.

MWA induced robust immune response and up-regulated TIGIT in multi-immune cell types. Therefore, we combined MWA and anti-TIGIT treatment and assessed the antitumor effect of such a combination strategy. Mice with MC38 colon cancer were divided into four groups as follows: the control group, which was treated with an isotype control antibody; the MWA group, which was treated with MWA and an isotype control antibody; the TIGIT group, which was treated with anti-TIGIT; and the MWA plus TIGIT group, which was treated with MWA and anti-TIGIT. We observed delayed tumor growth in the MWA, TIGIT, and MWA plus TIGIT groups compared with the control group. We also observed prolonged survival in the MWA, anti-TIGIT, and MWA plus anti-TIGIT groups (**Figure 2A**). Furthermore, the survival was much longer in the MWA plus anti-TIGIT treatment group compared with the other treatment groups (**Figure 2B**). To understand the synergistic antitumor effect induced by the combination of MWA and anti-TIGIT treatment, we performed a multi-color flow cytometric analysis of TILs in mice treated under the four above-mentioned conditions. We found that the frequencies of CD45^+^ TILs and CD8^+^ TILs were higher in the MWA, TIGIT, and MWA plus TIGIT groups compared with the control group (**Figures 2C, E**). Additionally, in the MWA plus TIGIT group, the frequencies of CD45^+^ TILs and CD8^+^ TILs were higher compared with the MWA or TIGIT alone group (**Figures 2C, E**). Furthermore, the numbers of CD4^+^ T cells and NK cells were increased in the mice receiving MWA alone and combination therapy (**Figures 2D, F**). However, no significant differences in the frequency of Tregs among the four groups were observed (**Figure 2G**).

### The Combination of MWA and Anti-TIGIT Treatment Enhances CD8^+^ T Cell Responses in Distant Tumors

**Figure 3:**
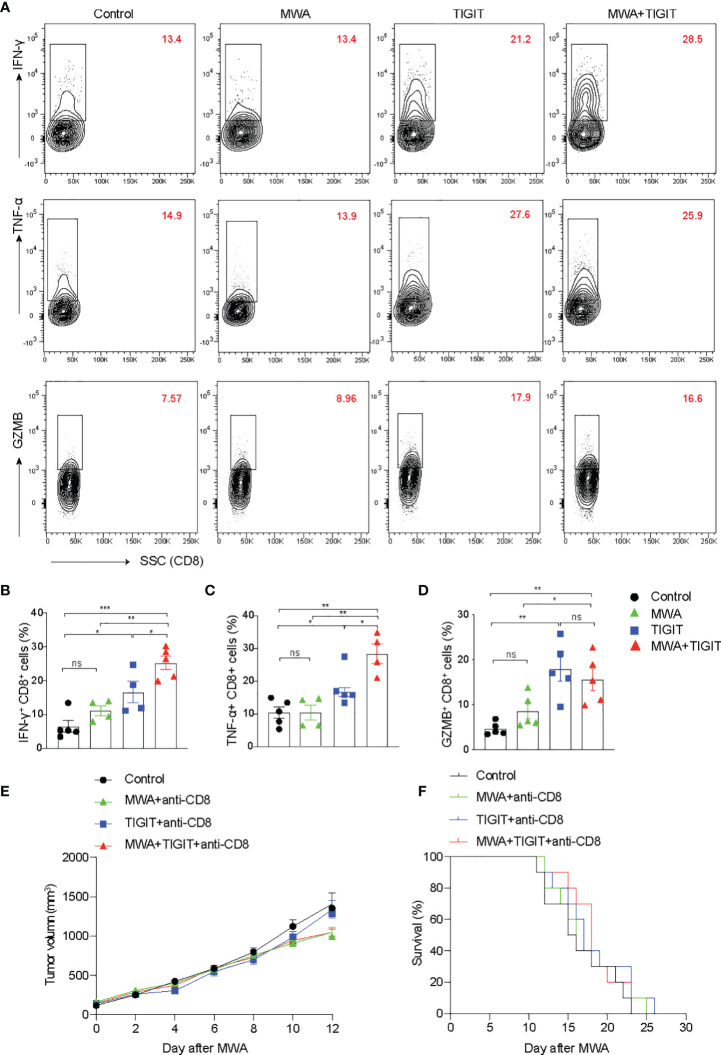
The combination of MWA and anti-TIGIT treatment enhances the CD8^+^ T responses in distant tumors. **(A–D)** A total of 3×10^6^ MC38 cells were subcutaneously inoculated into the bilateral flanks of C57BL/6 mice (n=4-5). The MC38 tumor-bearing mouse models were treated with MWA, anti-TIGIT antibody, and MWA plus anti-TIGIT antibody, as described in **Figure 2**. **(A)** Representative flow cytometry plots of IFN-γ, TNF-α and GZMB expressions in CD8^+^ T cells. **(B–D)** Quantitative analysis of the percentages of IFN-γ, TNF-α and GZMB expressions in CD8^+^ T cells. Data were presented as the mean ± SEM, n=4-5, ns (not significant, *P*>0.05), * *P*<0.05, ** *P*<0.01, *** *P*<0.001, and **** *P*<0.0001 according to the one-way ANOVA test. **(E, F)** For the depletion of CD8^+^ T cells, the mice were administered 250 µg anti-CD8 antibodies starting from 1 day before MWA and subsequently every 3 days after MWA. **(E)** The tumor size on the left blank after MWA was measured every 2 days after MWA. **(F)** Kaplan–Meier survival curves were shown. Data were presented as the mean ± SEM, ns (not significant, *P*>0.05), * *P*<0.05, ** *P*<0.01, and *** *P*<0.001 according to the two-way ANOVA test and the log-rank test.

We next examined the T cell response under different treatment conditions. We found that the production of interferon-γ (IFN-γ), tumor necrosis factor-α (TNF-α), and granzyme B (GZMB) in CD8^+^ T cells in the MWA group were similar to those in the control group (**Figures 3A, B**). In addition, we found that the expressions of IFN-γ, TNF-α, and GZMB were higher in CD8^+^ T cells from the TIGIT or MWA plus TIGIT group compared with the control group (**Figures 3A–D**). Furthermore, the frequencies of IFN-γ^+^ and TNF-α^+^ CD8^+^ T cells were higher in the MWA plus anti-TIGIT group compared with the MWA or TIGIT group. Consistently, after depletion of CD8^+^ T cells, we found that the tumor growth and the survival were similar among these groups (**Figures 3E, F**). These data suggested that blockade of TIGIT enhanced MWA-induced antitumor immune responses, and this effect was mediated by CD8^+^ T cells.

### The Combination of MWA and Anti-TIGIT Treatment Reverses the Exhaustion of CD8^+^ T Cells in the TME

**Figure 4:**
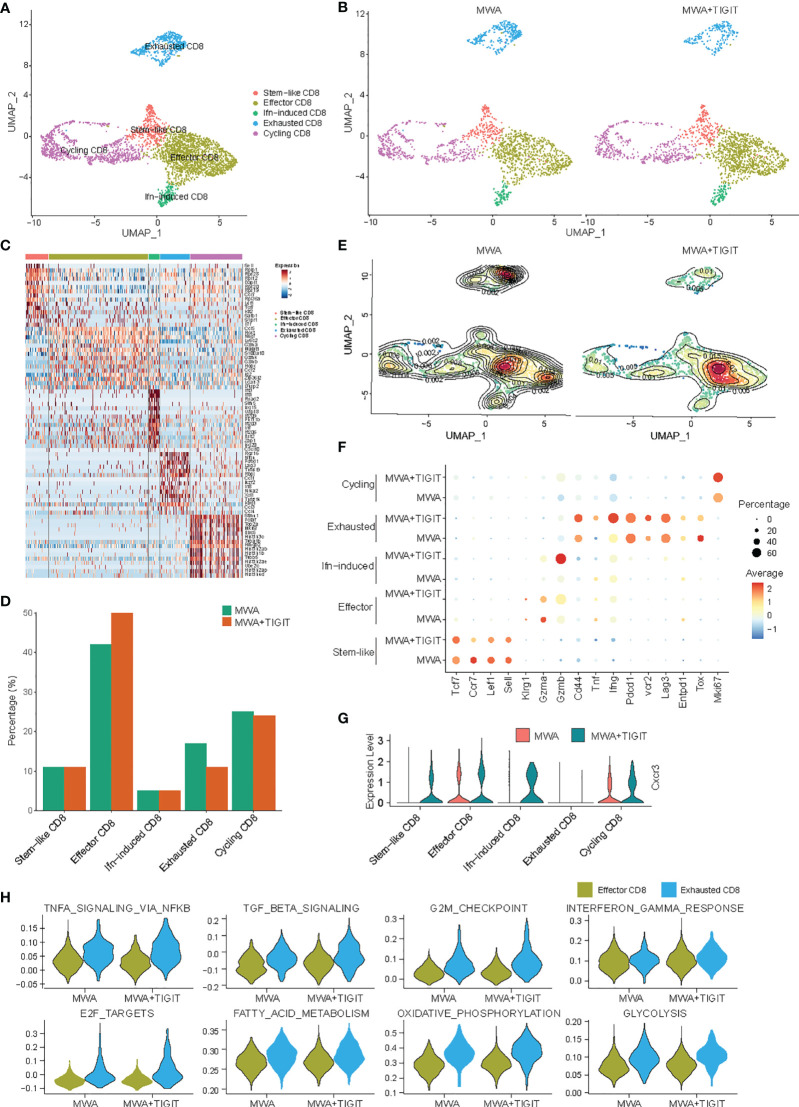
The combination of MWA and anti-TIGIT treatment reverses the exhaustion of CD8^+^ T cells in the TME. MC38 tumor-bearing mouse models were established and treated with MWA or MWA plus anti-TIGIT antibody, and CD45^+^ TILs were then purified using FACS and subjected to scRNA-seq analysis. **(A)** UMAP analysis showed that CD8^+^ TILs were classified into stem-like, interferon-induced, effector, exhausted and cycling subpopulations. **(B)** UMAP analysis of different subpopulations of CD8^+^ TILs in the MWA group or MWA plus TIGIT group. **(C)** Heatmap displaying marker genes expressed in different subpopulations of CD8^+^ TILs. **(D)** Percentages of different subpopulations of CD8^+^ TILs in the MWA group or MWA plus TIGIT group. **(E)** Contour map showing different subpopulations of CD8^+^ TILs in the MWA group or MWA plus TIGIT group. **(F)** Selected DEGs were identified in different sub-populations of CD8^+^ TILs in the two groups. **(G)** Violin plot showing the expression of the chemokine receptor CXCR3 in different subpopulations of CD8^+^ TILs in the MWA group or MWA plus TIGIT group. **(H)** Violin plot showing the selected pathways regulated by effector CD8^+^ T cells and exhausted CD8^+^ T cells in the MWA group or MWA plus TIGIT group.

To assess the different roles of antitumor T cell-mediated immune responses between the MWA group and the MWA plus anti-TIGIT group, we analyzed scRNA-seq data for conventional T cell subsets. We found that the CD8^+^ TILs were generally classified into five subpopulations based on the expressions of marker genes, including stem-like CD8^+^ T cells, effector CD8^+^ T cells, interferon-induced CD8^+^ T cells, exhausted CD8^+^ T cells, and cycling CD8^+^ T cells (**Figures 4A–E**). The addition of anti-TIGIT treatment to the MWA regimen increased the frequency of effector CD8^+^ T cells and decreased the frequency of exhausted CD8^+^ T cells in the TME (**Figures 4D, E**). However, there were almost no significant differences in the frequencies of stem-like CD8^+^ T cells, interferon-induced CD8^+^ T cells, and cycling CD8^+^ T cells (**Figures 4D, E**). An analysis of differentially expressed genes (DEGs) showed that the expression of markers of exhausted T cells (Pdcd1, Havcr2, and Lag-3) were high in the MWA plus TIGIT and MWA groups. However, CD44, TNF-α, and IFN-γ were up-regulated in exhausted T cells. Nevertheless, the expressions of effector T cell markers, such as GZMB, TNF, IFN-γ and CD44, exhibited almost no significant differences in expression between the MWA plus anti-TIGIT and MWA groups (**Figure 4F**). Based on these data, the combination of MWA and anti-TIGIT treatment reversed the exhaustion of CD8^+^ T cells and increased the frequency of effector CD8^+^ T cells in the TME. Furthermore, the chemokine receptor CXCR3 is expressed on CD8^+^ T cells and plays a role in T cell migration into tumors (32, 33). We found that CXCR3 was up-regulated in stem-like CD8^+^ T cells, effector CD8^+^ T cells, interferon-induced CD8^+^ T cells, and cycling CD8^+^ T cells (**Figure 4G**). We further studied the functions of effector CD8^+^ T cells and exhausted CD8^+^ T cells between the MWA group and MWA plus TIGIT group by comparing the activities of related pathways. We found that pathways associated with TNF-α, TGF-β, oxidative phosphorylation, and glycolysis responses were upregulated in the effector CD8^+^ T cells in the MWA plus TIGIT group, whereas NF-κB-mediated TNF-α signaling, TGF-β signaling, G2/M checkpoint, IFN-γ, fatty-acid metabolism, oxidative phosphorylation, and glycolysis pathways were upregulated in the exhausted CD8^+^ T cells in the MWA plus TIGIT group (**Figure 4H**). These data indicated that the combination of MWA and anti-TIGIT treatment increased the quantities of effector CD8^+^ T cells and reversed the function of exhausted CD8^+^ T cells.

### The Combination of MWA and Anti-TIGIT Treatment Reshapes Myeloid Cells in the TME

**Figure 5:**
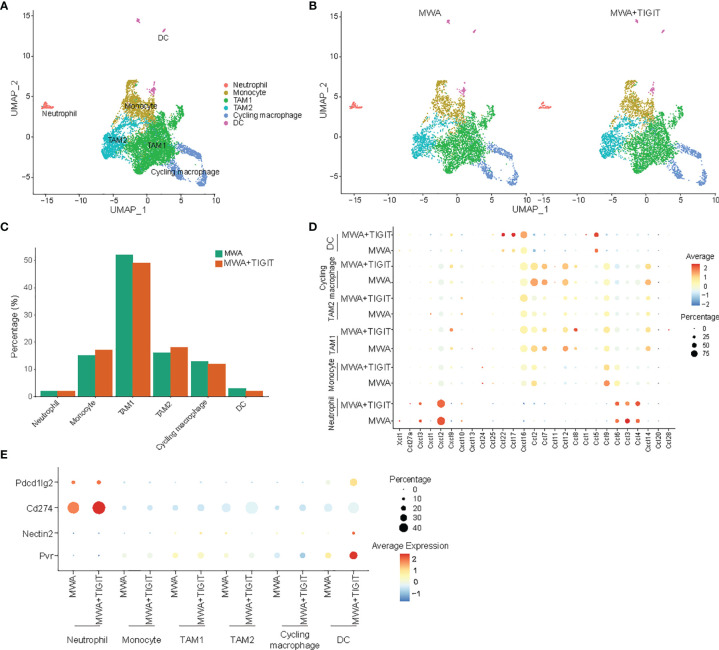
The combination of MWA and anti-TIGIT treatment reshapes myeloid cells in the TME. MC38 tumor-bearing mouse models were established and treated with MWA and MWA plus anti-TIGIT antibodies, and then CD45^+^ TILs were purified using FACS and subjected to scRNA-seq analysis. **(A)** UMAP analysis showed that tumor-infiltrating myeloid cells were classified into neutrophils, monocytes, TAM1s, TAM2s, cycling TAMs, macrophages and DCs. **(B)** UMAP analysis showed the distribution of different subpopulations of myeloid cells in tumors from the MWA group or MWA plus TIGIT group. **(C)** Bar plot showing the percentages of different subpopulations of myeloid cells in MC38 tumors from the MWA group or MWA plus TIGIT group. **(D)** Dot plot showing selected chemokines in different subpopulations of myeloid cells in MC38 tumors from the MWA group or MWA plus TIGIT group. **(E)** Dot plot showing the ligands of TIGIT and PD-1 expressed on myeloid cells in MC38 tumors from the MWA group or MWA plus TIGIT group.

Subsequently, we performed a unified manifold approximation and projection (UMAP) analysis of scRNA-seq data to examine myeloid cell populations. Notably, we did not detect significant differences in the major myeloid cell populations in the TME between the MWA group and the MWA plus TIGIT group (**Figures 5A–C**). We then examined the expressions of chemokines in neutrophils, monocytes, tumor-associated macrophages (TAM)1s, TAM2s, cycling macrophages and dendritic cells (DCs). CXCL9 and CXCL10 are chemokine ligands of CXCR3, which differ from other chemokines due to their ability to restrain tumor growth and enhance antitumor immunity (34, 35). Our data revealed that the production of CXCL9 by TAM1s was higher in the MWA plus TIGIT group. CCL5 plays a role in the chemoattraction and activation of immune cells, and it enhances tumor infiltration by immune cells (36). Co-expression of CCL5 and CXCL9 is considered as a marker of immunoreactive tumors that are associated with prolonged survival and responsiveness to PD-1 blockade (37). In the present study, we found that CCL5 was up-regulated synergistically with CXCL9 in DCs in the MWA plus TIGIT group (**Figure 5D**). CCL2 mediates tumor cell metastasis by binding to CCR2 on tumor cells and induces the expression of matrix metalloproteinase-9 (MMP-9) to increase the invasiveness of tumor cells (38). CCL2 can also shift Th2 cell regulation in the direction of immunosuppression by participating in inducing the differentiation and polarization of T lymphocytes (39). As an effective chemokine of TAMs, CCL2 indirectly promotes the invasion and metastasis of tumor cells through TAM chemotaxis (40). CCL7 binds to CCR2 and has a similar function to that of CCL2 (41). Our data showed that the expressions of CCL2 and CCL7 were decreased after the combination treatment of MWA and TIGIT blockade (**Figure 5D**). This result indicated that the combination therapy could reduce the migration and the recruitment of immunosuppressive cells to the TME.

Notably, CXCL16 plays an ambiguous role in cancer. CXCL16 inhibits liver metastasis *via* the recruitment of CXCR6-expressing T cells and invariant NKT (iNKT) cells and improves the survival of cancer patients (42, 43). Additionally, Veinotte et al. have found that CXCL16-positive DCs can enhance iNKT cell-dependent IFN-γ production and tumor control (44). However, the role of CXCL16 in cancer is complex, as transmembrane CXCL16 may promote antitumor responses, whereas soluble CXCL16 may contribute to tumor progression (45). Hojo et al. have found that the expression of CXCL16 by the tumor cells enhances the recruitment of TILs, thereby leading to a better prognosis in CRC (46). Consistently, we found that CXCL16 was upregulated in DCs in the MWA plus TIGIT group (**Figure 5D**). TIGIT has multiple ligands, namely, CD155 (PVR), CD112 (nectin-2), and CD113 (nectin-3) and our study revealed that PVR and nectin-2 were upregulated on DCs of the MWA plus TIGIT group. Additionally, PD-L1 (CD274), and PD-L2 (Pdcd1lg2) were upregulated in the MWA plus TIGIT group (**Figure 5E**). These data indicated that the TIGIT/PVR or TIGIT/nectin-2 axis, in addition to the PD-L1/PD-1 axis, was involved in the reduced efficacy of MWA.

### The Combination of MWA and Anti-TIGIT Treatment Alters the Cell-Cell Communication Across All Immune Subsets

**Figure 6:**
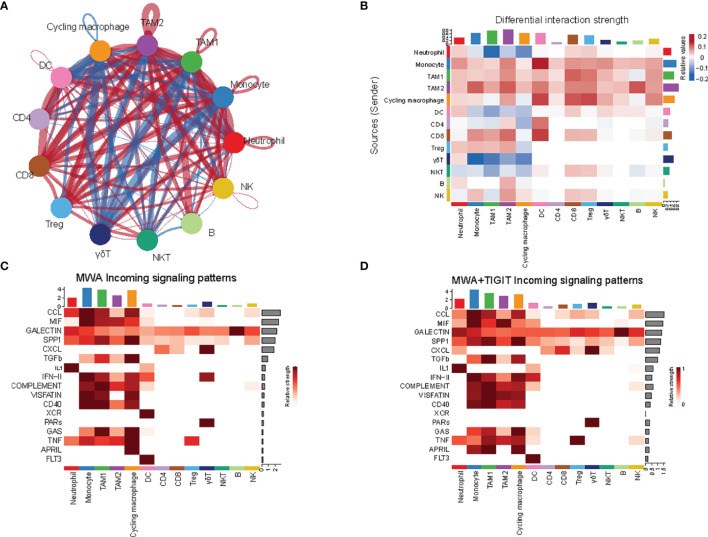
The combination of MWA and anti-TIGIT treatment alters the cell-cell communication across all immune subsets. **(A, B)** The cell-cell communication among immune cell populations between the MWA group and MWA plus TIGIT group. Blue represents a strong interaction among the immune cell populations of the MWA group, and red represents a strong interaction among the immune cell populations of the MWA plus TIGIT group. **(A)** Ring diagram. **(B)** Heatmap. **(C, D)** Heatmap showing the differences in receptor signaling pathways among immune cell populations between the MWA group and the MWA plus TIGIT group. **(C)** Receptor signaling pathways among immune cell populations in the MWA group. **(D)** Receptor signaling pathways among immune cell populations in the MWA plus TIGIT group.

To fully analyze differences in the communication among immune cell populations between the MWA group and the MWA plus TIGIT group, we applied the CellChat package, which combines social network analysis, pattern recognition and multiple learning methods to quantitatively describe and compare the inferred intercellular communication networks (47). In the MWA plus TIGIT group, the interaction between myeloid cells was significantly weakened, and the interaction between T cells, including CD4, CD8, and Treg cells, was significantly enhanced (**Figures 6A, B**), indicating that the changes induced by the addition of anti-TIGIT treatment to MWA might be related to the anti-TIGIT-mediated blockade of the inhibitory signal on the T cells. We further analyzed the differences in receptor signaling pathways among immune cell populations between the MWA group and the MWA plus TIGIT group. We found that the CCL and CXCL signals in CD8^+^ T cells were significantly increased in the MWA plus TIGIT group compared with the MWA group (**Figures 6C, D**).

## Discussion

Combination immunotherapy based on ICIs has achieved significant success in the treatment of many types of cancers (48, 49). According to combination immunotherapy, ablation in combination with immunotherapy has shown efficacy in some preclinical and clinical studies. Shi et al. have previously reported that RFA induces T cell-mediated immune responses in distant tumors (10). However, RFA does not always induce prolonged immune responses in the later stages, while the PD-1/PD-L1 axis plays an important role in the suppression of RFA responses, and the combination of PD-1 blockade and RFA alters the immunosuppression (10). In addition, RFA does not prevent tumor recurrence in some patients, suggesting the existence of other mechanisms underlying immune suppression. In the present study, we found that the expression of TIGIT was up-regulated after MWA, indicating that the expression of TIGIT, which was up-regulated as an immunosuppressive signal after MWA, was an important implication for the combination therapy. TIGIT, an immune inhibitory receptor, is co-expressed with PD-1. Therefore, TIGIT may participate in the immunosuppressive effect of MWA. Furthermore, we found that the combination of MWA and anti-TIGIT treatment prolonged the survival of mice with MC38 colon cancer and inhibited tumor growth. We also found that the MWA increased the infiltration of CD4^+^ T, CD8^+^ T, and CD45^+^ T cells in the TME, and the MWA plus anti-TIGIT treatment further increased the infiltration of these cells.

Strategies to reactivate exhausted antitumor responses using PD-1/PD-L1 or CTLA-4 blockade have been demonstrated effective in the clinic (50). However, not all the patients benefit from immunotherapy (51). These results indicate that multiple immune checkpoints are involved in immunosuppression. TIGIT blockade is a promising immunotherapy for cancer when combined with PD-1/PD-L1 blockade, while it does not significantly inhibit tumor growth when administrated alone (52). In the present study, we found that the anti-TIGIT treatment alone exerted limited effects on tumor growth and mouse survival in the mouse model with MC38. Notably, many ongoing clinical trials are attempting to assess the application of TIGIT blockade or TIGIT blockade in combination with anti-PD-1/PD-L1, anti-CTLA-4 or anti-LAG-3 antibodies in the treatments for many cancers (53). A rational combination of treatments that can induce antitumor immune responses and anti-TIGIT combination therapy should substantially increase the good responses of patients. Local therapies, such as RFA and MWA, may induce an immune response. Therefore, we combined MWA and TIGIT blockade and found that the combination therapy resulted in significantly reduced tumor growth and extended long-term survival. Furthermore, the combination treatment significantly improved the function of TILs, reversed the exhaustion of CD8^+^ T cells, and increased the frequency of effector CD8^+^ T cells in the TME. Moreover, our findings revealed that pathways associated with TNF-α, TGF-β, oxidative phosphorylation, and glycolysis responses were relatively up-regulated in the effector CD8^+^ T cells in the MWA plus TIGIT group, whereas NF-κB-mediated TNF-α signaling, TGF-β signaling, the G2/M checkpoint, IFN-γ, fatty-acid metabolism, oxidative phosphorylation, and glycolysis pathways were upregulated in the exhausted CD8^+^ T cells in the MWA plus TIGIT group. Consistent with our findings, a recent study has demonstrated that the combination of TIGIT blockade and anti-PD-Ll treatment synergistically enhances the function of CD8^+^ T cells (23). Moreover, ICIs, including anti-CTLA-4 and anti-PD-1/PD-L1 antibodies, have successfully reinvigorated TILs and benefited a large number of cancer patients (54). Therefore, enhancing the frequencies and function of effector CD8^+^ T cells and reversing the function of the exhausted CD8^+^ T cells are important in enhancing anti-tumor immunity.

Myeloid cells are important components of the TME and consist of various cellular subtypes. Myeloid cells are operationally divided into mononuclear and polymorphonuclear cells. The former cells include macrophages and DCs, and the latter cells include neutrophils, eosinophils, mast cells and basophils (55). The tumor recruits and modulates endogenous myeloid cells to TAMs, DCs, myeloid-derived suppressor cells (MDSCs) and neutrophils (TANs) to sustain an immunosuppressive environment (56). Based on the scRNA-seq results, by analyzing the myeloid cell population, we found that the percentages of neutrophils, monocytes, TAM1s, TAM2s, cycling macrophages and DCs were similar between the MWA group and MWA plus TIGIT group. Then we further examined the expressions of chemokines of myeloid cells. Our data revealed that the addition of TIGIT blockade to MWA upregulated the expressions of CXCL9 and CXCL10 in TAMs and the expression of their receptor CXCR3 in T cells, which might restrain tumor growth and enhance antitumor immunity. It is well known that CXCR3 is a chemokine receptor expressed on CD4^+^ T, CD8^+^ T, and NK cells, and it has three ligands, namely, CXCL9, CXCL10, and CXCL11 (57). CXCR3-CXCL9/CXCL10 chemokine axis is known for its tumor-inhibiting properties. Furthermore, our study also found that CCL5 was up-regulated synergistically with CXCL9 in DCs in the MWA plus TIGIT group in our study. Consistent with our conclusions, Chow et al. have found that the CXCR3-CXCL9 chemokine axis plays a more local role in promoting interactions between antitumor T cells and DCs present within the tumor during PD-1 therapy (58). A similar role for the CCL5/CXCL9 chemokine axis has been previously observed in a preclinical mouse model administered PD-1-based therapy (37). The chemokines CCL2 and CCL7, which are known as ligands of CCR2, promote the invasion and metastasis of tumor cells and induce immunosuppression (59). Based on our data, the expression of CCL2 and CCL7 levels were decreased after the combination treatment of MWA and TIGIT blockade. Consistent with our results, our previous study has found that the incomplete RFA is involved in rapid tumor progression and hinders PD-1 blockade immunotherapy due to the production of CCL2 by cancer cells (11). CXCL16-positive DCs enhance iNKT cell-dependent IFN-γ production and inhibit tumor growth (44). We detected higher percentages of CXCL16^+^ DCs in the MWA plus TIGIT group compared with the MWA group, indicating that the combination of TIGIT blockade and MWA induced immunoreactivity and enhanced tumor control.

In summary, the expression of TIGIT was increased after MWA, and the addition of anti-TIGIT therapy to MWA synergistically inhibited the growth of the tumor, increased the number of effector CD8^+^ T cells, and reversed the function of exhausted CD8^+^ T cells. Besides, the addition of anti-TIGIT therapy to MWA reshaped the myeloid cells by up-regulating CXCL9, CCL5 and CXCL16, and down-regulating CCL2 and CCL7, which reduced immunosuppression in the TME. Furthermore, the addition of anti-TIGIT therapy to MWA weakened the interaction between myeloid cells and significantly enhanced the interaction between T cells, indicating that the combination of anti-TIGIT treatment and MWA inhibited the inhibitory signals on the T cells in a synergistic manner. Collectively, the combination of TIGIT blockade and MWA could be used in the clinical practice to reprogram the TME toward an antitumor environment.

## Materials and Methods

### Cell Lines and Mice

The mouse colon cancer cell line MC38 was obtained from the Chinese Academy of Sciences, Shanghai Institutes for Biological Sciences. MC38 cells were maintained in DMEM (Gibco, Thermo Fisher Scientific, USA) supplemented with 10% (v/v) fetal bovine serum (FBS, Gibco, Thermo Fisher Scientific, USA), 100 U/mL penicillin, and 100 μg/mL streptomycin. Male C57BL/6 mice (6-8 weeks old) were purchased from and housed in a the specific-pathogen-free (SPF) facility at Cavens Laboratory Animals (Changzhou, China). All animal experiments were conducted according to protocols approved by the Ethics Committee of the Third Affiliated Hospital of Soochow University.

### Animal Models and *In Vivo* Treatment

MC38 (3×10^6^) cells were subcutaneously inoculated into the bilateral flanks of C57BL/6 mice. MWA was only carried out on the tumor on the right flank when the tumor volume reached approximately 300 mm^3^. MWA was conducted using an ablation electrode percutaneously inserted in the center of the tumor. The treatments were performed for 2 to 4 minutes at 70°C and 8 W. TIGIT blockade was administered intraperitoneally four times every 3 days starting 1 day after MWA. An anti-TIGIT mAb (Clone 1G9, BioXcell, USA) was administered at a dose of 200 μg per mouse per injection. For the depletion of CD8^+^ T cells, the mice were administrated 250 μg anti-CD8 antibodies (Clone 2.43, BioXcell, USA), starting from 1 day before MWA and subsequently every 3 days after MWA, and the mice belonging to the control group were administered control IgG. The diameters of the tumors on the left flank were measured every 2 days and the tumor volume was calculated using the formula as follows: V=Length x Width^2^/2.

### Flow Cytometry

Tumors were collected from the mice, minced into pieces smaller than 1 mm^3^, and digested with Liberase TL (REF 05401020001, Roche) and DNase I (REF 10104159001, Roche) at 37°C for 30 min. Subsequently, a serum-containing culture medium was added to terminate the digestion, and the pieces were ground and filtered through a 200-μm strainer to obtain a single-cell suspension. Anti-mouse antibodies were used to stain cells including antibodies against CD45 (Clone 30-F11), Ghost (Cell Signaling Technology), CD3 (Clone 17A2), CD4 (Clone GK1.5), CD8 (Clone 53-6.7), NK1.1 (Clone PK136), FOXP3 (Clone MF-14), and TIGIT (Clone IG9). For the measurement of intracellular cytokine levels, the cells were stimulated with PMA (50 ng/mL, Sigma-Aldrich), ionomycin (1 μg/mL, Sigma-Aldrich), and monensin (GolgiStop, BD Biosciences) at 37°C for 4 hours. After stimulation, the cells were stained with antibodies against surface markers, fixed, and permeabilized according to the manufacturer’s instructions provided by the Invitrogen Fixing/Permeabilization Solution kit. The fixed cells were stained using antibodies against IFN-γ (Clone XMG1.2), TNF-α (Clone MP6-XT22), and GZMB (Clone GB11). Data were acquired using a BD FACS Aria II flow cytometer and were analyzed using FlowJo software.

### ScRNA-Seq

Single-cell suspensions of the tumors were prepared as using the method described above. The cells were enriched using the CD45(TIL) Microbead Mouse Kit (Cat. No.: 130-110-618, Miltenyi Biotec, Lerden, the Netherlands) according to the magnetic-activated cell sorting (MACS) protocol and stained with the antibodies, Ghost Dye™ Violet 510 Viability Dye (Cell Signaling Technology) and Percp-Cy5.5-CD45 (Clone 30-F11) for FACS sorting. Approximately 1×10^5^ CD45^+^ cells/sample were sorted using a BD Aria II instrument. Based on the FACS analysis, single cells were sorted into flow tubes, and the cell viability was tested by determining the AOPI to ensure sufficient cell quality. Then, the cell suspension, which contained 300-600 living cells per microliter, as determined using CountStar, was loaded onto the chromium single-cell controller (10x Genomics) to generate single-cell gel beads in the emulsion according to the manufacturer’s instructions. Single-cell transcriptome amplification was performed with an S1000™ Touch Thermal Cycler (Bio-Rad) at 53°C for 45 min, followed by incubation at 85°C for 5 min, and holding at 4°C. The cDNA templates were generated and then amplified, and the quality was assessed using an Agilent 4200 instrument (performed by CapitalBio Technology, Beijing).

### ScRNA-Seq Data Processing

The newly generated scRNA-seq data obtained from 10X Genomics were aligned to the mm10 mouse reference genome and quantified using the Cell Ranger Single-Cell Software Suite. The preliminarily filtered data generated by Cell Ranger were used for a Seurat object created by the R package Seurat (version 3.2.3). Doublets were removed with the DoubletFinder package. Further quality control was applied to cells based on three metrics in a stepwise manner, including the total UMI count, the number of detected genes, and the proportion of mitochondrial gene count per cell. Specifically, cells with more than 5,000 UMI counts and 10% mitochondrial gene counts were filtered out.

### Integration of Multiple scRNA-Seq Data, Dimension Reduction and Unsupervised Clustering

Single-cell data were processed for dimension reduction and unsupervised clustering analysis by following the workflow in Seurat. Briefly, 2,000 highly variable genes were selected for downstream analysis by using the Find Variable Features function with the parameter “n features = 2000.” Subsequently, the Integrated Data function was used to integrate the data and create a new matrix with 3,000 features, in which the potential batch effect was regressed out. Principal component analysis (PCA) was performed on an integrated data matrix to reduce the dimensionality of the scRNA-seq dataset. Using the Elbow plot function of Seurat, the top 40 PCs were subjected to the downstream analysis. The main cell clusters were identified using the Find Clusters function in Seurat, with the resolution set as 0.1. Subsequently, the clusters were visualized by constructing 2D tSNE or UMAP plots. Conventional markers described in a previous study were used to categorize each cell into a known biological cell type.

### Differential Gene Expression Analysis

The EdgeR package (version 3.28.1) was used to select DEGs between different clusters. The raw data obtained from the Seurat object were normalized using TMM (trimmed mean of M-values) with the calcNormFactors function, and the dispersion of gene expression values was estimated using the estimateDisp function. The DEGs were selected using the Seurat package DotPlot function for visualization.

### Statistical Analysis

Statistical analyses were performed using Prism (GraphPad) V.8 software, and the data were presented as mean ± SEM. The two-tailed unpaired Student’s *t*-test was used to compare two groups and the ANOVA test was used for multiple comparisons. The survival of the mice was analyzed using the Kaplan-Meier method and calculated using the log-rank test. Significance levels were defined as ns (not significant, *P*>0.05), * *P*<0.05, ** *P*<0.01, *** *P*<0.001, and **** *P*<0.0001.

## Data Availability Statement

The datasets presented in this study can be found in online repositories. The names of the repository/repositories and accession number(s) can be found below: GEO database with accession number is GSE194209.

## Ethics Statement

The animal study was reviewed and approved by The Ethics Committee of the Third Affiliated Hospital of Soochow University.

## Author Contributions

LC, CW and XZ conceived the project and supervised the research. HH and YC performed the bioinformatics, computational analysis, and biological interpretation of data. YC, HH and LC performed the statistical analysis. YC and HH performed the statistical analysis. YC, WX, and RC performed the tumor models and animal experiments. YC, YL, and YTL carried out the flow cytometry analysis and flow sorting experiments. YC and YZ carried out the cell culture. CW, LC, XZ, and YC prepared the manuscript. All authors have read the article and approved the final version of this manuscript.

## Funding

Funding was supported by the National Key R&D Plan (2018YFC1313400), the National Natural Science Foundation of China (82172689, 81902386, 81972869), the Natural Science Foundation of Jiangsu Province (BK20211065), China Postdoctoral Science Foundation (2021M700543, 2021M700547), High-Level Talents Project of Jiangsu Commission of Health (LGY2020034), the Applied Basic Research Foundation of Changzhou (CJ20190094) and Changzhou International Cooperation Project (CZ20210035).

## Conflict of Interest

The authors declare that the research was conducted in the absence of any commercial or financial relationships that could be construed as a potential conflict of interest.

## Publisher’s Note

All claims expressed in this article are solely those of the authors and do not necessarily represent those of their affiliated organizations, or those of the publisher, the editors and the reviewers. Any product that may be evaluated in this article, or claim that may be made by its manufacturer, is not guaranteed or endorsed by the publisher.

## Supplementary Material

Supplementary Figure 1 TIGIT expression changes in tumor procession. A total of 3×10^6^ MC38 cells were subcutaneously inoculated into the bilateral flanks of C57BL/6 mice (n=4). MWA was performed on the tumor on the right flank when the tumor volume reached approximately 300 mm^3^. **(A–D)** Representative flow cytometry plots of TIGIT expression in TILs, including CD4^+^ T, CD8^+^ T, NK cells and CD4^+^ Foxp3^+^ Tregs of untreated animals on day 0 (means control day 0), untreated animals on day 10 (means control day 10), and day 10 after MWA (MWA day 10). Data were presented as the mean ± SEM, ns (not significant, *P*>0.05), * *P*<0.05, ** *P*<0.01 according to the one-way ANOVA test.
