# Supplementary figures and images for: TIGIT Blockade Exerts Synergistic Effects on Microwave Ablation Against Cancer
Source: Front Immunol. 2022 Mar 7;13:832230. doi: 10.3389/fimmu.2022.832230 (PMC8935077; doi:10.3389/fimmu.2022.832230)

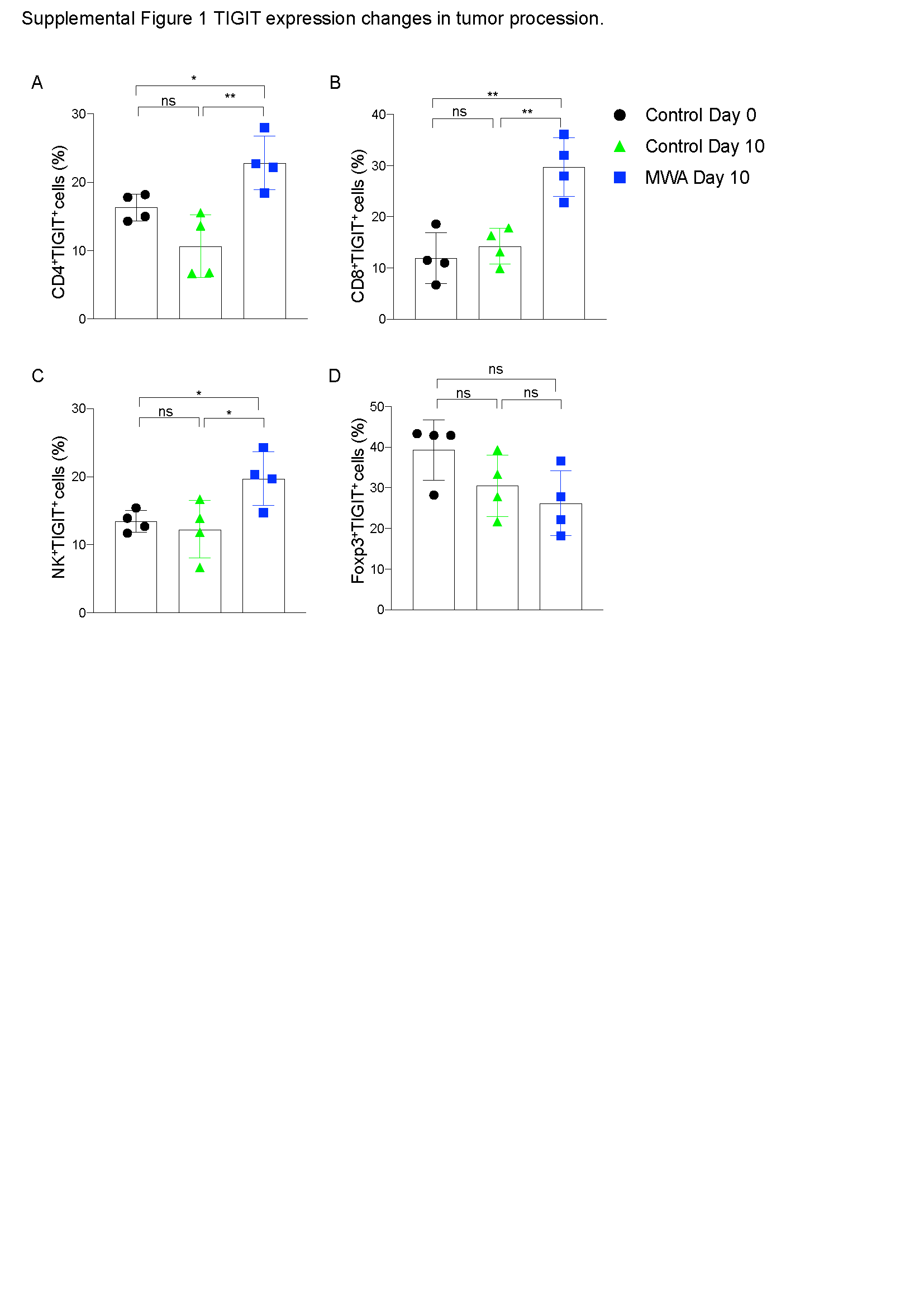

Supplement: Supplementary Figure 1 — TIGIT expression changes in tumor procession. A total of 3×10^6^ MC38 cells were subcutaneously inoculated into the bilateral flanks of C57BL/6 mice (n=4). MWA was performed on the tumor on the right flank when the tumor volume reached approximately 300 mm3. (A–D) Representative flow cytometry plots of TIGIT expression in TILs, including CD4+ T, CD8+ T, NK cells and CD4+ Foxp3+ Tregs of untreated animals on day 0 (means control day 0), untreated animals on day 10 (means control day 10), and day 10 after MWA (MWA day 10). Data were presented as the mean ± SEM, ns (not significant, P>0.05), * P<0.05, ** P<0.01 according to the one-way ANOVA test. [file Image_1.tiff]
